# A Micro-Platinum Wire Biosensor for Fast and Selective Detection of Alanine Aminotransferase

**DOI:** 10.3390/s16060767

**Published:** 2016-05-26

**Authors:** Tran Nguyen Thanh Thuy, Tina T.-C. Tseng

**Affiliations:** Department of Chemical Engineering, National Taiwan University of Science and Technology, Taipei 10607, Taiwan; M10206808@mail.ntust.edu.tw

**Keywords:** alanine aminotransferase, microelectrode, amperometric, glutamate oxidase, biosensor

## Abstract

In this study, a miniaturized biosensor based on permselective polymer layers (overoxidized polypyrrole (Ppy) and Nafion^®^) modified and enzyme (glutamate oxidase (GlutOx)) immobilized micro-platinum wire electrode for the detection of alanine aminotransferase (ALT) was fabricated. The proposed ALT biosensor was measured electrochemically by constant potential amperometry at +0.7 V *vs.* Ag/AgCl. The ALT biosensor provides fast response time (~5 s) and superior selectivity towards ALT against both negatively and positively charged species (e.g*.*, ascorbic acid (AA) and dopamine (DA), respectively). The detection range of the ALT biosensor is found to be 10–900 U/L which covers the range of normal ALT levels presented in the serum and the detection limit and sensitivity are found to be 8.48 U/L and 0.059 nA/(U/L·mm^2^) (*N* = 10), respectively. We also found that one-day storage of the ALT biosensor at −20 °C right after the sensor being fabricated can enhance the sensor sensitivity (1.74 times higher than that of the sensor stored at 4 °C). The ALT biosensor is stable after eight weeks of storage at −20 °C. The sensor was tested in spiked ALT samples (ALT activities: 20, 200, 400, and 900 U/L) and reasonable recoveries (70%~107%) were obtained.

## 1. Introduction

Liver diseases can often lead to failing of normal liver functions (e.g., metabolic function, digestion, protein synthesis, detoxification, *etc.*). The development of advanced biosensing techniques plays an important role in the diagnosis of liver diseases and tracing liver conditions and therefore, these diseases may be controlled or treated if identified early. Common liver function tests often contain measurements of bilirubin, albumin, transferases (e.g., alanine aminotransferase (ALT), aspartate aminotransferase (AST), γ-glutamyltransferase (GGT), *etc.*), and other enzymes (e.g., alkaline phosphatase (AP), lactate dehydrogenase (LDH), *etc.*) [1]. Among them, aminotransferases (ALT and AST) are the most useful measures of hepatic injury. On the other hand, when hepatocytes are damaged, the ALT level can increase up to 50 times greater than normal; therefore, compared to AST, the ALT level is more liver specific [2]. ALT can be found in cardiac muscles, skeletal muscles, kidneys, and mainly in the liver. The normal ALT concentration in the blood is ~5–35 U/L (the upper limit threshold of ALT level is generally ~40 U/L) [2,3]. Abnormalities of ALT activity in the serum may be associated with some chronic liver diseases such as alcoholic liver disease, chronic hepatitis B or C, hepatocellular carcinoma, *etc.* [1]

A variety of analytical approaches have been used to determine the ALT level in liver function tests, such as colorimetry [4,5,6], spectrophotometry [7,8], chemiluminescence [9], chromatography [10], fluorescence [11,12,13], electrochemical techniques [14,15,16,17,18], *etc.* When techniques like fluorescence, chromatography, or chemiluminescence are used, careful conductions of tests are required since tests could be affected by environmental factors (e.g., pH, light intensity, temperature, ionic strength, interactions in solutions, *etc.*) easily [19] and therefore result in low sensitivity for measuring ALT [2]. Spectrophotometry is the most routinely used method in the clinical laboratory which can provide precise and accurate detection of ALT; however, performing spectrophotometric measurements usually consumes large quantities of expensive reagents and requires cumbersome instruments and time-consuming incubation steps [3,20]. Recently, increased attention has been drawn to electrochemical techniques, including differential pulse voltammetry [21], chronoamperometry [22], cyclic voltammetry [23], constant potential amperometry [3,14,16,17,18,24,25,26,27,28,29], *etc.*, for the detection of ALT; in general, they provide fast response time and high sensitivity for measuring ALT; besides, they require only compact instruments and straightforward operations.

Different materials including gold (Au) [21,22], palladium (Pd) [28], graphite [23], platinum (Pt) [3,18,25,26,27], and other materials [14,17,24] for the fabrication of ALT biosensors were reported. Among them, Pt is more stable and inert [29,30] and shows better electrooxidation properties for hydrogen peroxide (H_2_O_2_), the product of some enzymatic reactions catalyzed by oxidases in presence of water and oxygen; therefore, Pt is a superior electrode material for fabricating biosensors using oxidases as biorecognition elements. In general, electrodes with small dimensions are preferred since the cost of sensor fabrication as well as the volume of tested samples can be reduced. To increase the sensitivity and selectivity of ALT biosensors, different enzymes working as biorecognition elements have been used to modify the electrode surface, including pyruvate oxidase (PyOx) [14,16,24], lactate dehydrogenase (LDH) [21], glutamate oxidase (GlutOx) [3,17,18,25,26,27,28], *etc.* In fact, GlutOx-based ALT biosensors have much higher storage stability and simpler fabrication process; besides, they do not require cofactors during measurements, whereas PyOx-based ALT biosensors require the addition of thiamine pyrophosphate and Mg^2+^ for testing [17,18]. Different modification layers including cellulose acetate [18] and Nafion^®^ [28] have been used during the fabrication of ALT biosensors to eliminate interference from the negatively charged ascorbic acid (AA) and uric acid (UA) presented in testing samples [18,23,27,28]; however, there are few study reporting on ALT biosensors which can reject both positively and negatively charged interferents.

**Figure 1:**
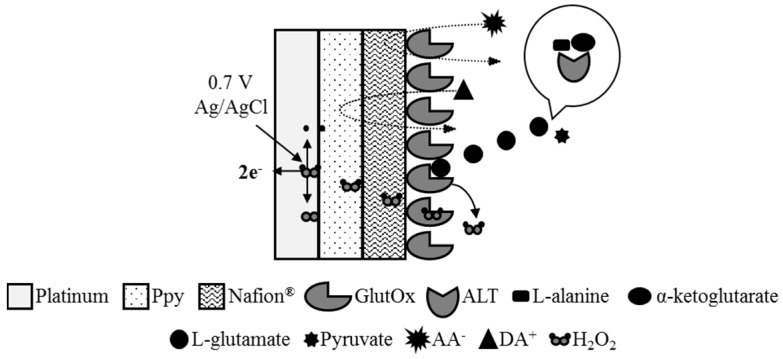
The schematic diagram showing the working principle of the ALT biosensor.

In this study, micro-Pt wire is chosen to be the base (*i.e.*, the working electrode) for the fabrication of the ALT biosensor. The biocatalytic reaction involved in the process of measuring ALT on the ALT biosensor is based on the enzymatic reaction of the target ALT producing L-glutamate and pyruvate in presence of L-alanine and α-ketoglutarate (Equation (1)); further oxidation of L-glutamate by glutamate oxidase (GlutOx) incorporated on the ALT biosensor yields H_2_O_2_, α-ketoglutarate, and ammonia (Equation (2)). The overall working principle of the ALT biosensor that we proposed is shown in Figure 1. The surface of the Pt working electrode was modified with the overoxidized Ppy layer in order to exclude electroactive interferents, such as ascorbic acid (AA) and dopamine (DA), and then the Nafion^®^ layer in order to reject anionic interferents [31,32] for enhancing the selectivity of the ALT biosensor. GlutOx was immobilized on the electrode atop those permselective modification layers for oxidizing L-glutamate produced from the previous enzymatic reaction (Equation (1)) and generating H_2_O_2_. The generated H_2_O_2_ is a small and neutral molecule which can permeate through permselective layers and reach the electrode surface. The electrooxidation of H_2_O_2_ occurs on the electrode surface when a constant potential (0.7 V *vs.* Ag/AgCl) applies (Equation (3)). The measured current signals can be correlated to ALT levels by calibration. In this work, a fast, small, and economic ALT biosensor is proposed for assisting the development of a convenient diagnosing technique for liver diseases.

(1) $$L-\text{alanine }+\;\alpha -\text{ketoglutarate}\overset{\text{ALT}}{\to }L-\text{glutamate }+\text{ pyruvate}$$

(2) $$L-\text{glutamate }+{\text{ O}}_{2}\;\overset{\text{GlutOx}}{\to }\;\alpha -\text{ketogularate }+{\text{ NH}}_{3}\;+{\text{ H}}_{2}O_{2}\;$$

(3) $$H_{2}O_{2}\to O_{2}+2H^{+}\;+\;2e^{-}\;$$

## 2. Materials and Methods

### 2.1. Materials

Pyrrole, L-alanine, α-ketoglutaric acid sodium salt, and 3-hydroxytyramine hydrochloride (dopamine hydrochloride, DA) were purchased from ACROS Organics™ (Bridgewater, NJ, USA). Nafion^®^ (perfluorinated ion exchange resin, 5 wt % in solution in lower aliphatic alcohols/H_2_O mix) was purchased from Aldrich (Milwaukee, WI, USA). Bovine serum albumin (BSA, ~66 kDa, purity > 98%, lyophilized powder), alanine aminotransferase (ALT, EC 2.6.1.2, from porcine heart, activity: ≥75 U/mg protein), and glutaraldehyde (GAH) were purchase from Sigma-Aldrich (St. Louis, MO, USA). L-glutamate oxidase (GlutOx, EC 1.4.3.11) was from US Biological (Marblehead, MA, USA). L-(+)-ascorbic acid (AA) was from Enzo^®^ (Farmingdale, NY, USA). Hydrogen peroxide (30% *w*/*v*) was from Panreac (Barcelona, Spain). All other chemicals were of analytical grade. Sodium phosphate buffer (PBS, pH 7.4) was composed of 50 mM sodium phosphate (dibasic) and 100 mM sodium chloride. The perfluoroalkoxy (PFA) coated platinum wire (inner diameter: 50.8 µm) used for preparing the working electrode was obtained from A-M Systems (Sequim, WA, USA). Ag/AgCl glass-bodied reference electrodes with 3 M NaCl electrolyte and platinum wire (diameter: 0.5 mm) auxiliary electrodes were purchased from ALS Co., Ltd. (Tokyo, Japan). The Ag wire (diameter: 203 µm) used for preparing the Ag/AgCl wire reference electrode was obtained from RoHS (Taipei, Taiwan).

### 2.2. Instrumentation

The electrochemical preparation of ALT biosensors and electrochemical measurements were conducted using a versatile multichannel potentiostat (model VSP 300, Bio-Logic SAS, Claix, France) in a three-electrode configuration consisting of a Pt wire (inner diameter: 50.8 µm) working electrode, a Pt wire (diameter: 0.5 mm) auxiliary electrode, and a reference electrode consisting of a glass-enclosed Ag/AgCl wire in 3 M NaCl solution or a Ag/AgCl wire (inner diameter: 203 µm), respectively. All potentials are reported *vs.* the Ag/AgCl reference electrode. The Ag/AgCl wire reference electrode was prepared by immersing the Ag wire (inner diameter: 203 µm) in 0.1 M HCl solution and then allowing the electrolysis reaction to occur using a regular AA battery (1.5 V) to form AgCl on the anode (*i.e.*, the Ag wire). Field emission scanning electron microscope (model JSM-7001F, JEOL, Peabody, MA, USA) was used for magnifying the surface of ALT biosensors.

### 2.3. Preparation of ALT Biosensors

The Pt wire microelectrode was prepared by stripping two ends of a piece of 1.5 cm Pt wire (inner diameter: 50.8 µm) covered by PFA to reveal 2.5 mm bare Pt wire on each end. Then, one end (the connection end) was soldered with a piece of 8 cm long copper wire (AWG = 30) and the solder knot was covered with the epoxy glue; the other end of the exposed Pt will be used as the working electrode for the preparation of ALT biosensors. Once the epoxy glue was dried, the electrode was ready to use. Right before use, the Pt working electrode was cleaned by sonicating with isopropyl alcohol.

For the construction of permselective polymer layers of ALT biosensors, the Ppy film was electrodeposited on the surface of Pt wire working electrodes by cyclic voltammetry method (0.2–1.2 V, 26 cycles, scan rate 20 mV/s) in 20 mM pyrrole solution in PBS. Then, these electrodes were dip-coated with Nafion^®^ and baked at 180 °C (3 min); the dip-coating and baking process was repeated two more times. The Ppy film was used to reject common positively charged interferents, such as DA, while the Nafion^®^ layer was used to reject common negatively charged interferents, such as AA. These permselective polymer layers would contribute to increase the selectivity of ALT biosensors towards the analyte against interferents and prevent the false positive current responses from interferents.

**Figure 2:**
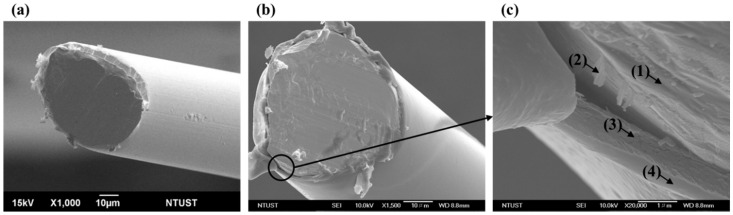
The cross-sectional structure of (**a**) the bare Pt wire electrode (×1000); (**b**) the ALT biosensor (×1500); and (**c**) the ALT biosensor (×20,000); layer (**1**): Bare Pt wire; layer (**2**): Overoxidased Ppy; layer (**3**): Nafion^®^; and layer (**4**): Immobilized enzyme layer.

The enzyme layer was constructed atop permselective polymer layers by dip-coating. The enzyme solution for deposition was prepared by mixing the GlutOx solution (250 U/mL) and the BSA solution (150 mM BSA and 53 mM GAH in DIW) in 1 to 1 volumetric ratio. The electrode was dip-coated with the enzyme solution for 90 times. The resulting ALT biosensors were stored at −20 °C overnight prior to use. The cross-sectional structure of the bare Pt wire electrode and the ALT biosensor were demonstrated in Figure 2. Approximate thickness of the Ppy layer, the Nafion layer, and the enzyme layer were ~0.01 µm, ~0.6 µm, and ~0.1 µm estimated from the SEM picture.

### 2.4. Calibration of ALT Biosensors by Method 1

**Figure 3:**
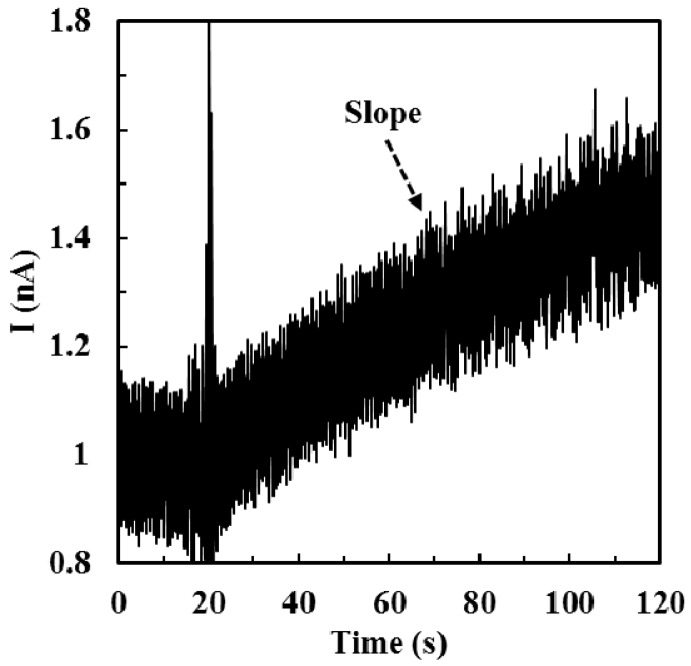
The typical I-t curve when testing ALT biosensors by *Method 1*. The injection timing of the ALT solution is at 20 s.

The calibration measurements were conducted by constant potential amperometry at 0.7 V and the current responses corresponding to different ALT activities *vs.* time were recorded independently. In *Method 1*, the testing solution was a 2.5 mL stirring PBS with ALT substrates (100 mM L-alanine and 10 mM α-ketoglutaric acid). The purchased ALT solid (1.61 mg, 124 U/mg solid) was diluted with 1 mL DIW to make 200 U/mL ALT stock solution; then, the stock solution was divided into 20 tubes (50 µL per tube) and stored at −20 °C. Prior to use, the ALT stock solution was warmed up to the room temperature and diluted again with 150 µL DIW to make 50 U/mL ALT solution. 2 µL, 10 µL, 20 µL, and 45 µL of the ALT solution (50 U/L) were injected into testing solutions directly and independently to make solutions with different ALT activities (40 U/L, 200 U/L, 400 U/L, and 900 U/L, respectively) and the corresponding current responses were recorded independently. The typical I-t curve obtained from testing ALT biosensors by *Method 1* was shown in Figure 3. The slope obtained was calculated within 60 s (*i.e.*, from 30th s to 90th s). The mean slope value of 0 U/L ALT was determined by calculating the average stable background current before each injection of ALT. The calibration curve was established by plotting slopes of current responses *vs.* ALT activities.

### 2.5. Calibration of ALT Biosensors by Method 2

The calibration measurements were conducted by constant potential amperometry at 0.7 V and the current responses corresponding to different ALT activities *vs.* time were recorded in a plot. In *Method 2*, the starting calibration solution was a 2 mL stirring PBS with ALT substrates (100 mM L-alanine and 10 mM α-ketoglutaric acid). The 50 U/L ALT solution was prepared as mentioned in Section 2.4. To prepare ALT calibrators, the ALT solution (50 U/mL) were mixed with substrate solutions (100 mM L-alanine and 10 mM α-ketoglutaric acid) to make eight ALT calibrators (ALT activities ranging from 0.21 U/mL, 0.23 U/mL, 0.48 U/mL, 0.76 U/mL, 0.82 U/mL, 5.3 U/mL, 8.4 U/mL, to 9 U/mL). The ALT-substrate mixture was prepared every 30 s (from lower to higher ALT activities) and let each ALT-substrate mixture react for 10 min before injecting it into the testing solution (PBS with 100 mM L-alanine and 10 mM α-ketoglutaric acid) for the electrochemical measurement. The current response of the stirring testing solution (2 mL solution without ALT) was first recorded for 30 s and the current response of the blank was obtained. After the 10 min-reaction of the first ALT-substrate mixture (calibrator **1**), 100 µL of the calibrator was injected into the 2 mL testing solution and the corresponding current response of 10 U/L ALT was recorded for another 30 s. The same procedure was followed for the electrochemical measurements of calibrator **2** to calibrator **8** and current responses of final ALT activities increased from 20 U/L, 40 U/L, 70 U/L, 100 U/L, 300 U/L, 600 U/L, to 900 U/L were recorded. The calibration curve obtained by *Method 2* was established by plotting current responses *vs.* ALT activities.

### 2.6. Determination of ALT Activities from Spiked Samples

Spiked ALT samples with known activities were tested and the measured ALT activities were compared to the actual known ALT activities. The setup and recording of electrochemical measurements for determining ALT activities from spiked samples was similar to that of calibration measurements. In *Method 1*, current responses of spiked ALT solutions corresponding to different known ALT activities (20 U/L, 200 U/L, 400 U/L, and 900 U/L) were measured by the same procedure as mentioned in Section 2.4. Based on the calibration curve obtained from calibration measurements of ALT biosensors tested by *Method 1*, activities of spiked ALT samples (*x*) can be calculated.

**Figure 4:**
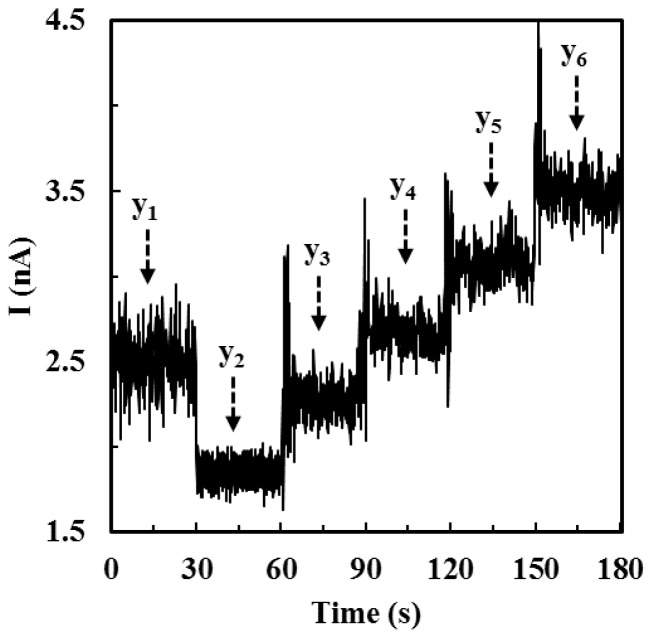
The typical I-t curve when testing ALT biosensors in spiked samples by *Method 2*. The measured current signals were represented by *y*_4_~*y*_6_.

In *Method 2*, the starting solution was a 3 mL stirring spiked ALT samples in PBS with substrates (100 mM L-alanine and 10 mM α-ketoglutaric acid). In this experiment, four individual spiked ALT samples with different ALT activities (20 U/L, 200 U/L, 400 U/L, and 900 U/L) were prepared. To prepare these spiked ALT samples, the ALT stock solution was mixed with the substrate solution (100 mM L-alanine and 10 mM α-ketoglutaric acid) for allowing ALT to react with substrates for 10 min. One minute before the reaction ended, the first ALT calibration mixture (6.1 U/mL) was prepared in PBS with substrates and the other three ALT calibration mixtures (6.3 U/mL, 6.5 U/mL, and 6.7 U/mL) were prepared every 30 s sequentially. Once the spiked ALT sample had reacted for 10 min, its corresponding current response (*y*_1_ in Figure 4) was recorded for 30 s. Subsequently, 3 mL of substrate solution (without ALT) was injected into the testing solution and the current response of the diluted testing solution (*y*_2_ in Figure 4) was recorded for another 30 s. Afterwards, 100 µL the first ALT calibration mixture was added to the testing solution and its corresponding current response (*y*_3_ in Figure 4) was recorded for 30 s. At this moment, the ALT activity in the testing solution was (100 + 0.5*x*) U/L where *x* is the activity of the spiked ALT sample. The similar procedure was repeated for the addition of the second to the forth calibration mixture and current responses (*y*_4_~*y*_6_ in Figure 4) corresponding to (200 + 0.5*x*) U/L, (300 + 0.5*x*) U/L, and (400 + 0.5*x*) U/L were recorded. Once the I-t curve (Figure 4) was obtained, the slope (*a*) of the linear calibration equation acquired from the calibration data (y_3_~y_6_ *vs.* (100 + 0.5*x*) U/L~ (400 + 0.5*x*) U/L) could be calculated. This slope *a* was plugged into to Equations (4) and (5). After solving Equations (4) and (5) simultaneously, the activity of the spiked sample could be determined based on our ALT biosensors tested by *Method 2*. The same procedure was repeated for determining the activities of other spiked ALT samples (200 U/L, 400 U/L, and 900 U/L).

(4) $$y_{1}=ax+b$$

(5) $$y_{2}=\frac{ax}{2}+b$$

## 3. Results and Discussion

### 3.1. Calibration of ALT Biosensors

#### 3.1.1. Sensitivity, Detection Limit, Sampling Time, and Detection Range of ALT Biosensors Tested by *Method 1*

**Figure 5:**
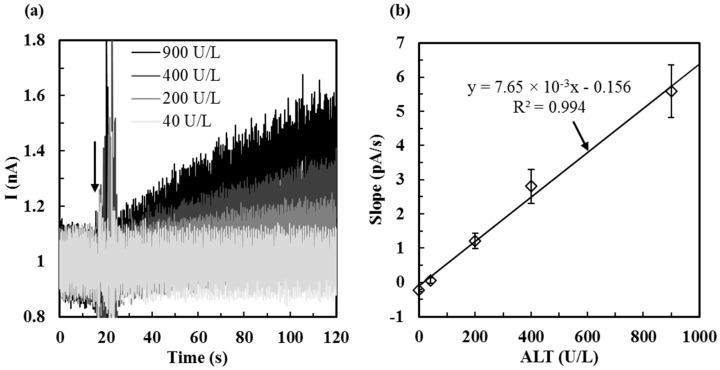
(**a**) ALT current responses corresponding to ALT activities (from 40 U/L, 200 U/L, 400 U/L, to 900 U/L) tested by *Method 1* after one day of storage at −20 °C. The arrows indicate the timing of ALT addition; (**b**) The calibration curve of ALT biosensors (*N* = 10) tested by *Method 1*.

For those ALT biosensors tested by *Method 1*, they were stored at −20 °C for one day before testing. ALT current responses corresponding to ALT activities (from 40 U/L, 200 U/L, 400 U/L, to 900 U/L) tested by *Method 1* were shown in Figure 5a. The detection range is from 40 to 900 U/L and the range covers the normal ALT concentration presented in the serum and beyond. In *Method 1*, the ALT current responses increased as the enzymatic reaction continued and the signal was recorded for 60 s (*i.e.*, the sampling time). The calibration curve of ALT biosensors tested by *Method 1* was established by plotting the slope of each ALT current response (pA/s) *vs.* ALT activities (U/L) as shown in Figure 5b. The sensitivity was defined by the slope of the calibration curve divided by the electrode area (0.401 mm^2^) of the biosensor. Based on the calibration curve in Figure 5b, the biosensor sensitivity was calculated to be 1.908 × 10^−5^ ± 6.739 × 10^−6^ nA/(s·U/L·mm^2^). This sensitivity was higher than that presented by Kihara *et al.* [16], but not as high as that proposed by Cooper *et al.* [17] and Chang *et al.* [28]. It is possibly due to the thick permselective layers coated on our ALT biosensors which reduce the biosensor sensitivity. To increase the sensor sensitivity, possible solutions may involve the usage of proper mediators including ferrocene derivatives, quinine derivatives, and other organic dyes which have been used in the fabrication of biosensors for the detection of glucose [33] or H_2_O_2_ [34] where the redox reaction of the mediator is coupled with enzymatic oxidation to mediate electron transfer between the active center of enzyme and electrode and thus enhances the electron transfer. However, our ALT biosensors have much better selectivity towards ALT against both positively charged interferents DA and negatively charged interferents AA (further discussion of the effect of interference will be discussed in Section 3.2). Detection limit was 15.9 U/L and was determined by the calibration equation (Equation (6)) obtained in Figure 5), where *x* is the limit of detection and *y* is the mean slope of 0 U/L ALT added with two times deviation. Besides, the size of our ALT biosensors were much smaller than that of other ALT biosensors proposed by others (~125 times, ~4.5 times, and ~10 times smaller than that proposed by Kihara *et al.* [16], Cooper *et al.* [17], and Chang *et al.* [28], respectively) which means the fabrication cost of our ALT biosensors and the reagent volume for testing can be reduced considerably.

(6) $$y=7.65\times 10^{-3}x-0.156$$

#### 3.1.2. Sensitivity, Detection Limit, Sampling Time, and Detection Range of ALT Biosensors Tested by *Method 2*

**Figure 6:**
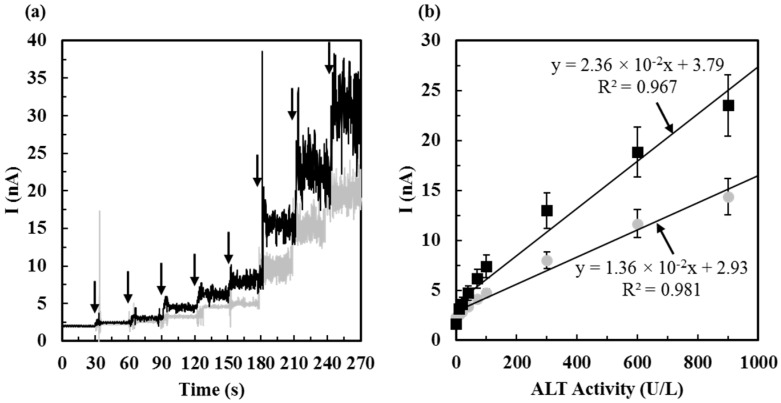
(**a**) ALT current responses corresponding to ALT activities (from 0 U/L, 10 U/L, 20 U/L, 40 U/L, 70 U/L, 100 U/L, 300 U/L, 600 U/L, to 900 U/L) of biosensors tested by *Method 2* after one day of storage at 4 °C (━ *gray line*) and −20 °C (━ *black line*). The arrows indicate the timing of ALT additions; (**b**) Calibration curves of ALT biosensors (*N* = 10) tested by *Method 2* after one day of storage in the refrigerator at 4 °C (●) and −20 °C (■).

To determine sensitivities of ALT biosensors tested by *Method 2*, ALT current responses corresponding to ALT activities (from 0 U/L, 10 U/L, 20 U/L, 40 U/L, 70 U/L, 100 U/L, 300 U/L, 600 U/L, to 900 U/L) were recorded (Figure 6a) and the sensitivity was calculated based on the slope of the calibration curve (Figure 6b). It was found that the storage temperature was also an important factor which can affect the sensitivity of ALT biosensors. Once fabricated, ALT biosensors were tested after one day of storage at −20 °C and 4 °C. It was shown that ALT biosensors prepared after one day of storage at −20 °C have higher sensitivity and a lower detection limit (0.059 ± 0.029 nA/(U/L·mm^2^) and 8.48 U/L, respectively) compared to that prepared after one day of storage at 4 °C (0.034 ± 0.016 nA/(U/L·mm^2^) and 14.74 U/L, respectively). The sensitivity was defined by the slope of the calibration curve divided by the electrode area (0.401 mm^2^) of the biosensor and the detection limit was defined by the ALT concentration corresponding to two times the level of noise. The sensitivity and detection limit of the biosensor prepared by storing at −20 °C was 1.74 times higher than that prepared by storing at 4 °C. This result suggests that a lower storage temperature (−20 °C) during the preparation of ALT biosensors may preserve higher enzyme activity of glutamate oxidase immobilized on the biosensor and therefore, the biosensors have higher sensitivity towards glutamate, the product of the ALT enzymatic reaction. Besides, our ALT biosensors tested by *Method 2* have fast response time (~5 s) defined as the time required reaching 95% of the steady-state sensing current. Jamal *et al.* [3] proposed an ALT biosensor with response time ~36 times longer than ours, resulting in a long testing interval (8000 s), but the sensitivity and detection limit of their sensors were 2.6 times higher and 2.6 lower than those of sensors proposed in this study. Song *et al.* [26] proposed an ALT biosensor in the micro fluidic system with impressive sensitivity (2.69 nA/(U/L·mm^2^)) and detection limit (1.3 U/L); however, the detection range of their sensor only covered from 1.3 to 250 U/L and no information regarding the effect of interference was provided, while severe liver-damaged patients can have ALT levels elevated to 240–960 U/L [3]. The detection range of ALT biosensors tested by *Method 2* in this work is from 10 to 900 U/L and the range covers the normal ALT concentration presented in the serum and beyond.

### 3.2. Effect of Interference

**Figure 7:**
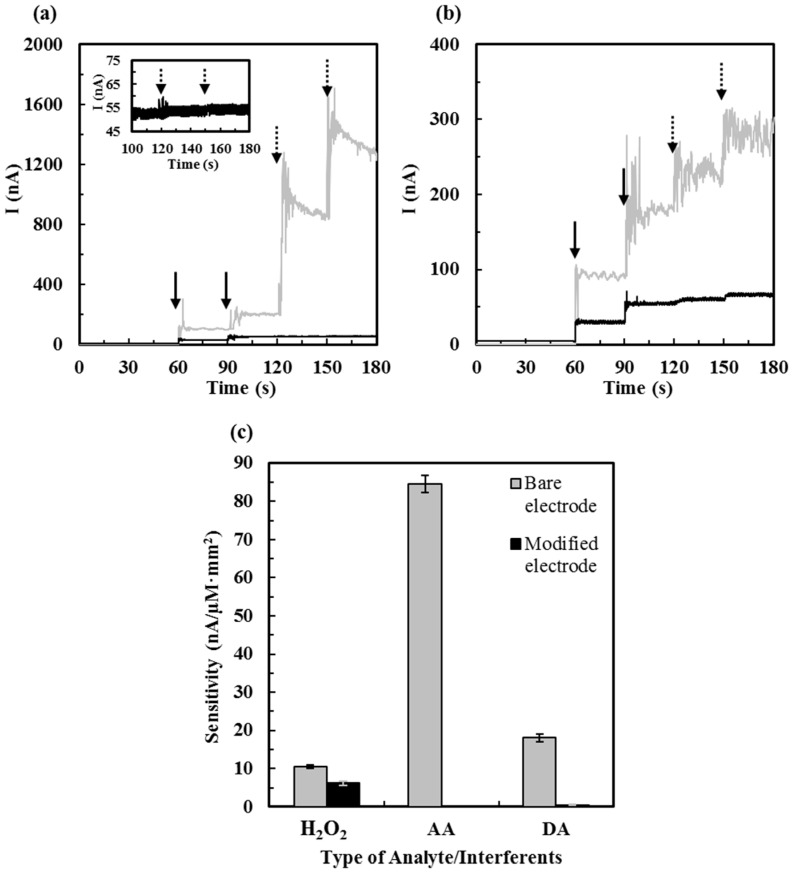
The effect of interference tested on bare Pt electrodes and ALT biosensors. (**a**) Current responses upon sequential additions of 20 µM and 40 µM H_2_O_2_ (the injection of H_2_O_2_ is indicated by the *solid black arrow*) and 250 µM and 500 µM AA (the injection of AA is indicated by the *dashed black arrow*) tested on bare Pt electrodes (━ *gray line*) and ALT biosensors (━ *black line*). The inset plot shows lower current range; (**b**) Current responses upon sequential additions of 20 µM and 40 µM H_2_O_2_ (the injection of H_2_O_2_ is indicated by the *solid black arrow*) and 5 µM and 10 µM DA (the injection of DA is indicated by the *dashed black arrow*) tested on bare Pt electrodes (━ *gray line*) and ALT biosensors (━ *black line*); (**c**) Comparison of interferent current responses tested on bare Pt electrodes and ALT biosensors (*N* = 5).

For recent studies working on ALT biosensors, only several of them have studies on the effect of interference from negatively charged AA and UA at low concentrations [18,23,27,28] and few of them reported the effect of interference from cations. To increase the selectivity of ALT biosensors, the electrode surface was coated with permselective polymer layers overoxidized Ppy and Nafion^®^ for hindering charged interferents. The schematic structure and the SEM image of these coatings was shown in Figure 1 and Figure 2c, respectively. In this experiment, positively charged dopamine (DA) and negatively charged ascorbic acid (AA) were used as exemplary interferent species. ALT biosensors and bare Pt electrodes were tested with 20 µM H_2_O_2_, 40 µM H_2_O_2_, 250 µM AA, and 500 µM AA, sequentially (Figure 7a), and with 20 µM H_2_O_2_, 40 µM H_2_O_2_, 5 µM DA, and 10 µM DA, sequentially (Figure 7b). Almost none of interferent current responses tested on ALT biosensors was found, but that tested on bare Pt electrodes showed excessively high AA and DA responses. The sensitivity of H_2_O_2_ tested on ALT biosensors also decreased (*i.e.*, 58.5%) suggesting that permselective layers modified on ALT biosensors could also lower the biosensor sensitivity; on the other hand, it was observed that permselective layers modified ALT biosensors have notably reduced noise signals. The interferent sensitivities of ALT biosensors towards AA and DA were reduced 3826 and 44.6 times, respectively, compared to those of bare Pt electrodes (see the bar chart shown in Figure 7c). Although these layers may decrease the flux of H_2_O_2_ reaching the Pt electrode surface resulting in lower current responses as shown in Figure 7c, the signal noises as well as the interferent currents decreased significantly as shown in Figure 7a,b. The concentration of AA presented in the cerebrospinal fluid is usually high (100–500 μM) [35,36] and the mean concentration of AA presented in the serum usually ranges from ~30–50 μM [35]. The concentration of DA ranges from ~0.01–1 µM in the extracellular fluid in the brain [36,37], but it was rarely found in the healthy blood [38,39,40]; however, DA may be presented in the blood at a high level if injected as a medication. Besides, the presence of some cationic pharmaceuticals (e.g., acetaminophen) may also interfere the sensing signal of ALT biosensors [18]. In summary, permselective polymer layers, overoxidized Ppy, and Nafion^®^ modified on ALT biosensors were shown to be able to reject common charged interferents AA and DA effectively. In Table 1, the figures of merit of recently reported electrochemical ALT biosensors are compared.

### 3.3. Stability of ALT Biosensors

**Figure 8:**
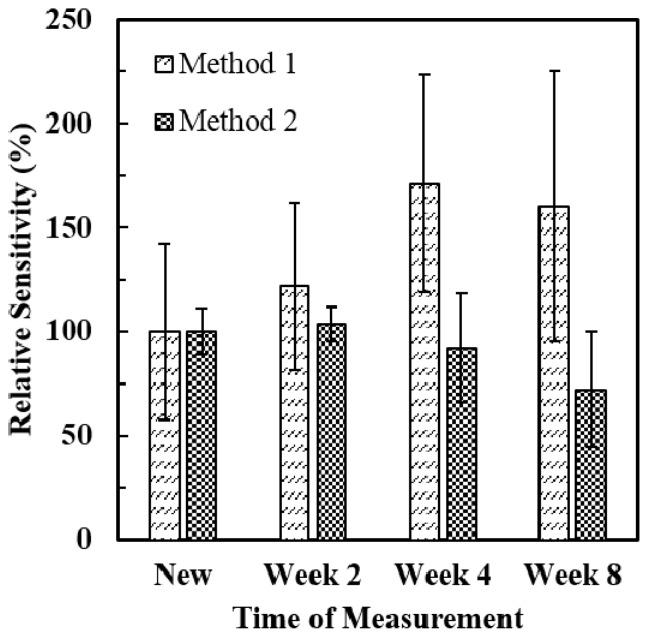
The storage stability of ALT biosensors tested by both methods. The relative sensitivity of ALT biosensors (*N* = 5) was plotted *vs.* time of measurement.

Many factors can affect the stability of enzyme-based amperometric biosensors; for example, the durability of electrodes, the method of enzyme immobilization, the method and condition of storage, *etc.* [25]. In this experiment, the stability of ALT biosensors was evaluated based on variations of sensor sensitivities during a period of time. Sensitivities of brand new ALT biosensors (*N* = 5) were measured on the first day (*New*), the 14th day (*Week 2*), the 28th day (*Week 4*), and the 56th day (*Week 8*) of storage (in a dessicant container at −20 °C) and the effect of storage time on the sensor sensitivity was investigated. In Figure 8, the relative sensitivity of the ALT biosensor was plotted *vs.* time of measurement to show the ability of the sensor to retain its initial sensitivity after a period of storage time. The relative sensitivity of the ALT biosensor was defined as the sensitivity of the sensor measured at certain time after storage divided by the initial sensitivity of the sensor. For ALT biosensors tested by *Method 1*, relative sensitivities of ALT biosensors were 100% (*New*), 122% (*Week 2*), 171% (*Week 4*), and 160% (*Week 8*), respectively. No decrease in sensor sensitivity was observed after eight weeks of storage. For ALT biosensors tested by *Method 2*, relative sensitivities of ALT biosensors were 100% (*New*), 104% (*Week 2*), 92% (*Week 4*), and 72% (*Week 8*), respectively. This result showed that ALT biosensors tested by *Method 2* can retain excellent sensitivities after storage for four weeks (relative sensitivity: 92%) and still have good sensitivities after storage for eight weeks (relative sensitivity: 72%). These results were comparable with Kihara *et al.* (80% after six months of storage at 4 °C, and 100% after one year of storage at −20 °C) [16], Cooper *et al.* (83% after 100 days of storage at 4 °C) [17], and Song *et al.* (85% after four months of storage at 4 °C) [26]. Overall, ALT biosensors tested by both methods can provide good storage stability up to two months. In addition, relative sensitivities of ALT biosensors were tested repeatedly in the 900 U/L ALT solution over two months to evaluate their reproducibility. When ALT biosensors were tested by *Method 1*, relative sensitivities of ALT biosensors were 100% (*New*), 139% (*Week 2*), 190% (*Week 4*), and 173% (*Week 8*). When ALT biosensors were tested by *Method 2*, their relative sensitivities were 100% (*New*), 94% (*Week 2*), 89% (*Week 4*), and 80% (*Week 8*). It showed that ALT biosensors tested in the 900 U/L ALT solution by *Method 2* provided better reproducibility over two months compared to those tested by *Method 1*.

### 3.4. Determination of ALT Activities in Spiked Samples

**Figure 9:**
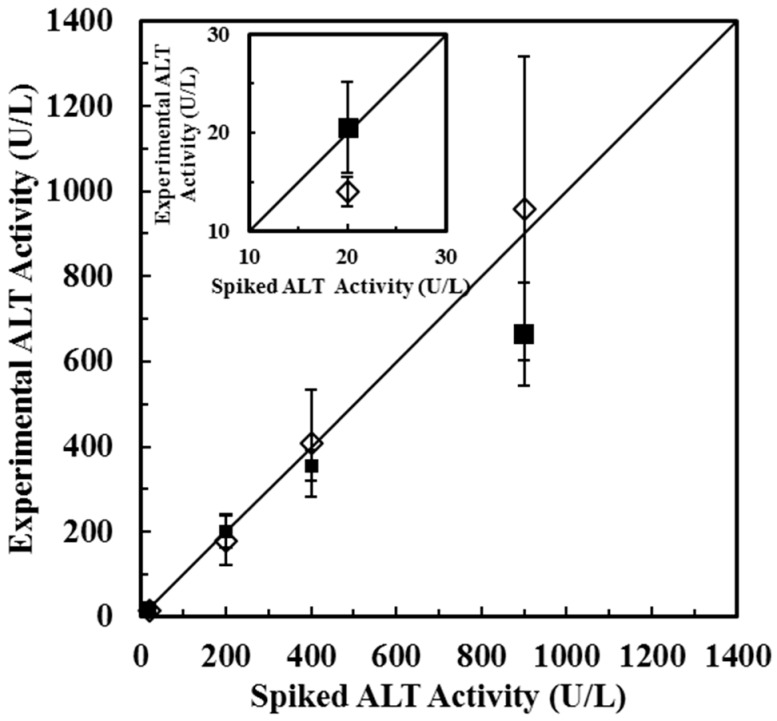
Experimental ALT activities measured by *Method 1* (◊) and *Method 2* (■) at spiked ALT activities: 20 U/L, 200 U/L, 400 U/L, and 900 U/L (*N* = 5). The inset plot shows lower activity range.

Spiked samples with different ALT activities (20 U/L, 200 U/L, 400 U/L, and 900 U/L) were tested by both *Method 1* and *Method 2*. The experimental ALT activities measured by *Method 1* were 14 U/L, 180 U/L, 408 U/L, and 959 U/L and the recoveries were found to be 70%, 90%, 102%, 107% corresponding to spiked ALT activities (20 U/L, 200 U/L, 400 U/L, and 900 U/L), respectively. The experimental ALT activities measured by *Method 2* were 21 U/L, 201 U/L, 355 U/L, and 663 U/L and the recoveries were found to be 103%, 101%, 89%, 74% corresponding to spiked ALT activities (20 U/L, 200 U/L, 400 U/L, and 900 U/L), respectively. The results of measured experimental ALT activities were shown in Figure 9. Based on the results, it suggested that *Method 1* provides better estimation for determining ALT activities at higher activity range (200~900 U/L), while *Method 2* provides better estimation for determining ALT activities at lower activity range (20~400 U/L).

## 4. Conclusions

A convenient, fast, and selective ALT biosensor based on platinum wire microelectrode modified by Ppy and Nafion^®^ and immobilized by GlutOx has been demonstrated successfully. Compared to other studies working on ALT biosensors, our sensors can provide the best selectivity against both negatively and positively charged interferents, the fastest response time (~5 s), good storage stability over eight weeks, good detection range (10–900 U/L), reasonable recoveries (70%–107%) in spiked ALT samples, and fair sensitivity (0.059 nA/(U/L·mm^2^)) and detection limit (8.48 U/L); in addition, the ultra-small size of the sensor allows economic production of the ALT biosensor as well as reducing the volume of testing reagents required. The proposed ALT biosensor may be used for assisting the development of a convenient diagnosing technique for liver diseases.

## Figures and Tables

**Table 1:** Recently reported electrochemical biosensors for the detection of alanine aminotransferase and their figures of merit.

No.	Electrode/size (mm^2^)	EC Method	Modification & Enzyme Layers	Selectivity	Response Time (s)	Sensitivity	LOD/range	Ref.
1	O_2_ electrode/490.9	CPA	Tf, porous AC & PyOx	NA	120	NA	0.5/0.5–180 U/L	[24]
2	Pt/50.3	CPA	PVC, CA & PyOx	NA	60	1.46 × 10^−5^ nA/(s·U/L·mm^2^)	5/5–1600 U/L	[16]
3	BIA membrane/28.3	CPA	PyOx only	NA	240	NA	3/6–30000 U/L	[14]
4	PAC/1.77	CPA	PC & GlutOx	NA	300	3.77 nA/(s·U/L·mm^2^)	5/5–500 U/L	[17]
5	Pt/0.05	CPA	CA, immobilon IA membrane, PC & GlutOx	AA, UA	300	NA	2/5–1200 U/L	[18]
6	Au/7.85 × 10^−3^	DPV	LDH only	NA	NA	0.122 nA/(s·U/L·mm^2^)	0.3/0.3–200 U/L	[21]
7	Au/19.63	ChA	SAM mediator-coated layer, anti-ALT Ab & PyOx	NA	NA	26.3 nA/(ng/mL)	0.01/0.01–1000 ng/mL	[22]
8	Pt/0.15	CPA	GlutOx only	NA	NA	NA	11/11–88 U/L	[25]
9	Pt/50.5	CPA	Nanoporous Si & GlutOx	NA	20	2.69 nA/(U/L·mm^2^)	1.3/1.3–250 U/L	[26]
10	Pt/0.15	CPA	GlutOx only	AA, UA	600	NA	9/ 9–250 U/L	[27]
11	Pd/4.8	CPA	Nf & GlutOx	AA, UA	120	2.07 × 10^−3^ nA/(s·U/L·mm^2^)	8/ 8–250 U/L	[28]
12	Pt/5.5	CPA	Nf, mediated medium & GlutOx	NA	180	0.153 nA/(U/L·mm^2^)	3.29/ 10–1000 U/L	[3]
13	Graphite/28.3	CV	P4AP & PyOx	AA, UA, Glut, Glu	200	NA	2.68×10^−5^/ 3 × 10^−5^–3 U/L	[23]
14	Pt/0.401	CPA	Ppy, Nf & GlutOx	AA, DA	60	1.908 × 10^−5^ nA/(s·U/L·mm^2^)	15.9/40–900 U/L	This work *^a^*
15	Pt/0.401	CPA	Ppy, Nf & GlutOx	AA, DA	5	0.059 nA/(U/L·mm^2^)	8.48/10–900 U/L	This work *^b^*

**Abbreviations:** Ab (antibody); AC (acetyl-cellulose); BIA (biodyne immunoaffinity); CA (cellulose acetate); ChA (chronoamperometry); CPA (constant potential amperometry); CV (cyclic voltammetry); DPV (differential pulse voltammetry; EC (electrochemical); Glu (glucose); Glut (glutamate); IA (immunoaffinity); NA (not available); Nf (Nafion); P4AP (poly(4-aminophenol)); PAC (platinized activated carbon); PC (polycarbonate); PVC (polyvinyl chloride); SAM (self-assembled monolayer); Tf (Teflon). **Superscriptions: **^a^ *Method 1*; ^b^ *Method 2.*
